# Dynamical response and noise limit of a parametrically pumped microcantilever sensor in a Phase-Locked Loop

**DOI:** 10.1038/s41598-023-29420-3

**Published:** 2023-02-07

**Authors:** João Mouro, Paolo Paoletti, Marco Sartore, Bruno Tiribilli

**Affiliations:** 1 Institute for Complex Systems, National Research Council (ISC-CNR), 50019 Sesto Fiorentino, Italy; 2 School of Engineering, University of Liverpool, Liverpool, L69 3GH UK; 3 Elbatech Srl, Via Roma, 10 - 57030 Marciana, Italy

**Keywords:** Applied physics, Statistical physics, thermodynamics and nonlinear dynamics, Sensors

## Abstract

We investigate the response of a digitally controlled and parametrically pumped microcantilever used for sensing in a Phase-Locked Loop (PLL). We develop an analytical model for its dynamical response and obtain an explicit dependence on the rheological parameters of the surrounding viscous medium. Linearization of this model allows to find improved responsivity to density variations in the case of parametric suppression. Experiments with a commercial microcantilever validate the model, but also reveal an increase of frequency noise in the PLL associated with the parametric gain and phase, which, in most cases, restricts the attainable limit of detection. The noise in open-loop is studied by measuring the random fluctuations of the noise-driven deflection of the microcantilever, and a model for the power spectral density of amplitude, phase and frequency noises is discussed and used to explain the frequency fluctuations in the closed-loop PLL. This work concludes that parametric pumping in a PLL does not improve the sensing performance in applications requiring detecting frequency shifts.

## Introduction

The quality factor of MEMS resonant sensors is a key parameter to determine the performance of many sensing applications. Techniques such as *Q*-control^1^, direct feedback or parametric pumping^2^ have been exploited as an effective way to modify the quality factor of the devices^3^. Parametric pumping and resonance are achieved by modulating a parameter of the resonant system, typically the spring constant, at twice the natural resonance frequency of the system. MEMS devices are good candidates to study the dynamic responses of parametric pumping and resonance^4^ due to their small dimensions and presence of mechanical/electrical nonlinearities.

The term parametric pumping is used when the modulation strength is below a certain threshold value and the response of the system, either amplified or suppressed, is stable. When the modulation gain exceeds this critical threshold, the system dynamics becomes unstable and enters in parametric resonance, exhibiting spontaneous oscillations close to submultiples of the resonance frequency^5^.

Several instability regions of parametric resonance have been observed in different systems^6^. For example, 28 modes were characterized in circular disks and proposed for energy harvesting^7^, while 14 different modes were used to study the properties of graphene membranes^8^. Since a parametric resonator only has two stable phases, these have been proposed for storing binary information in arrays^9^, for bit flip operations in mechanical computers^10^ or for amplifying signals through bifurcation control of coupled devices^11^. Parametric resonance was used to define new topologies of feedback loops^12,13^, for scanning microscopy and force spectroscopy^14^ or for amplifying the amplitude signal in liquids^15^.

Conversely, parametric pumping has been shown as an effective way to increase the amplitude and quality factor of oscillations^16–18^, but their range of applications is scarce. Recent works discuss the thermomechanical noise limits of parametric pumping in open-loop configuration^2,19^, and it has been suggested that the suppression regime is useful for applications requiring detecting frequency shifts^20^. A single study on the oscillation amplitude and stability of a parametrically pumped resonator in a closed-loop was reported^21^.

In this work we study the dynamical response of a PI-controlled and parametrically pumped resonator in a digital Phase-Locked Loop, to understand its sensing performance and noise limit. We (i) develop the PLL platform, model its dynamical response and use it to study the responsivity of the system and attainable limit of detection to density variations; (ii) observe that the frequency noise of the PLL increases with the parametric pump gain for all phases of the parametric pump, except one; (iii) observe that the noise-driven deflection of the parametrically pumped cantilever in open-loop configuration behaves similarly to the closed-loop PLL case; (iv) extend an existing analytical model for the amplitude and phase noises of parametrically pumped resonators in open-loop to explain the observed frequency noise in the PLL closed-loop configuration.

## Results

### Phase-Locked Loop platform

**Figure 1:**
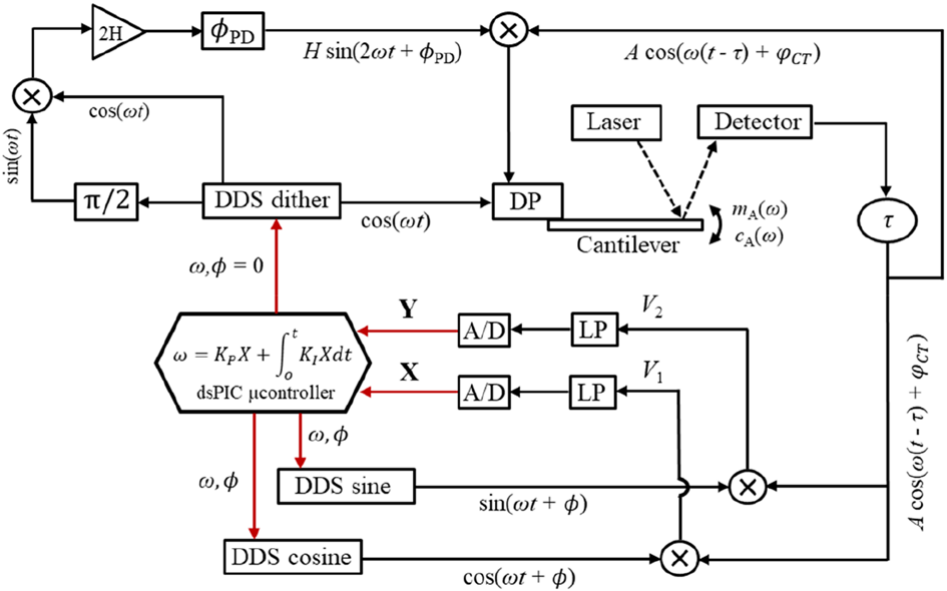
Schematic of the electrical signals throughout the developed PLL.

Figure 1 presents a schematic of the electrical signals throughout the platform developed in this work. It consists of a Phase-Locked Loop (PLL) with a parametrically pumped and digitally PI-controlled microcantilever. The microcantilever oscillates in a closed cell filled with a viscous medium and is excited by a Direct-Digital-Synthesized (DDS) signal applied to a dither piezo (DDS-dither). Its deflection is optically detected by a laser reflected to a four-quadrant detector and demodulated by two digital reference signals (DDS-sine and DDS-cosine), generated with an imposed phase $\phi$ with respect to the direct force term. The in-phase and quadrature components, *X* and *Y*, are low-pass filtered (LP), converted to digital and the *X*-signal fed to a microcontroller (dsPIC) to be used as the error parameter in a PI-controller which continuously adjusts the frequency *ω* of the synthetized signals.

The modulation of the spring constant of the cantilever at exactly twice the direct excitation frequency (degenerate case) is performed by a circuit in which the DDS-dither signal and a 90-degrees phase-shifted DDS-dither signal are multiplied (generating the signal at 2*ω*), amplified by a gain (2H), phase-shifted with respect to the direct driving force (${\phi }_{PD}$) and finally multiplied by the deflection of the microcantilever. This signal is added to the direct forcing term in the dither piezo.

Details on the electronic components of this system and a description of the dynamics and performance of the PLL can be found in “Materials and methods” section.

### Analytical model for the dynamical response of the cantilever in the PLL

The schematic shown in Fig. 1 is used to develop the analytical model describing the dynamical response of the microcantilever in the PLL (see the Supplementary Material for a detailed derivation).

The microcantilever is modelled as a parametrically pumped resonator^2,5^ in the degenerate case, including added mass and damping terms which depend explicitly on the rheological properties of the surrounding fluid^22,23^. The oscillation frequency of the closed PLL (imposed by the PI-controller) is obtained by analysing the steady-state of the system when the in-phase component is zero, *X* = 0 (corresponding to a perfectly tuned controller). These considerations make it possible to obtain an expression to calculate the frequency of oscillation $\omega$ of the PLL

1 $$- \omega \tau + {\text{arctg}}\left[ {\frac{{1 - \frac{{HQ_{R} }}{2k} {\text{sin}}\left( {\phi_{PD} } \right)}}{{2Q_{R} \left( {\frac{{\omega - \omega_{R} }}{{\omega_{R} }}} \right) + \frac{{HQ_{R} }}{2k} {\text{cos}}\left( {\phi_{PD} } \right)}}} \right] - \phi = - \left( {\frac{\pi }{2} + n\pi } \right),\,{\text{with}}\,n = 0, 1 ,2, \ldots$$

where the parameter $n$ describes the branch where the cantilever response can be found and depends on the delay around the loop $\tau$ (mostly due to the propagation of the acoustic waves from the piezo to the cantilever). The frequency of the oscillation $\omega$ calculated with Eq. (1) can then be used to determine the amplitude of oscillation with

2 $$A = \frac{{\left[ {\left( {2Q_{R} \left( {\frac{{\omega - \omega_{R} }}{{\omega_{R} }}} \right) + \frac{{HQ_{R} }}{2k} {\text{cos}}\left( {\phi_{PD} } \right)} \right)^{2} + \left( {1 - \frac{{HQ_{R} }}{2k} {\text{sin}}\left( {\phi_{PD} } \right)} \right)^{2} } \right]^{1/2} }}{{2Q_{R} \left( {\frac{{\omega - \omega_{R} }}{{\omega_{R} }}} \right)^{2} + 1 - \left( {\frac{{HQ_{R} }}{2k}} \right)^{2} }}.$$

In these expressions, $H$ is the parametric pump gain, $k$ is the cantilever spring constant, ${\phi }_{PD}$ is the phase between the parametric pump and the direct force term, and ${\omega }_{R}$ and ${Q}_{R}$ are the resonance frequency and quality factor of the resonator in the viscous fluid. The upper limit for the parametric pumping is given by ${H}_{th}=\frac{2k}{{Q}_{R}}$, above which the system loses stability and enters in parametric resonance^24^, from where the adimensional parametric pumping gain $\left|h\right|=\frac{H{Q}_{R}}{2k}$ is defined (taking values from 0 to 1).

The quality factor ${Q}_{R}$ contains information about the geometry of the cantilever and the rheological properties of the viscous medium

3 $$Q_{R} = \omega_{R} \left( {\frac{{m_{0} + m_{A} }}{{c_{0} + c_{A} }}} \right),$$

where the expressions for the resonance frequency in the viscous medium ${\omega }_{R}={\omega }_{0}{\left(1+\frac{{m}_{A}}{{m}_{0}}\right)}^{-\frac{1}{2}}$, the added mass ${m}_{\mathrm{A}}=\frac{\pi }{4}\rho L{W}^{2}\left({a}_{1}+\frac{{a}_{2}}{W}\sqrt{\frac{2\eta }{\rho \omega }}\right)$ and the added damping ${c}_{\mathrm{A}}=\frac{\pi }{4}\rho {LW}^{2}\omega \left(\frac{{b}_{1}}{W}\sqrt{\frac{2\eta }{\rho \omega }}+\frac{{b}_{2}}{{W}^{2}}\frac{2\eta }{\rho \omega }\right)$ are included. Here, $\rho$ and $\eta$ are the density and viscosity of the surrounding fluid, *a*_1_ = 1.0553, *a*_2_ = 3.7997, *b*_1_ = 3.8018, and *b*_2_ = 2.7364 are constants to describe the hydrodynamic function^23^, $L$ and $W$ are the length and width of the cantilever and ${\omega }_{0}$, ${m}_{0}$ and ${c}_{0}$ its natural resonance frequency in vacuum, total mass and intrinsic linear damping.

### Modelled responsivity to density variations in the PLL

The proposed analytical model can be used to study the responsivity of the system to density variations, defined as the change of oscillation frequency of the PLL induced by variations in the density of the fluid, or ${R}_{\rho }= \frac{\partial f}{\partial \rho }$. The nonlinear Eq. (1) can be linearized as

4 $$R_{\rho } \left( {\phi_{PD} ,\phi ,\eta ,\rho ,L,W,\tau ,m_{0} , \ldots } \right) = \frac{\partial f}{{\partial \rho }} \approx \left( {\frac{{f_{2} - f_{1} }}{{\rho_{2} - \rho_{1} }}} \right).$$

Equation (4) indicates the expected shift of frequency in real-time measurements of a process in which the gas density change by a small amount, around an initial working point. This method is general and can be used to study the responsivity to any other rheological or geometrical parameters^25^.

### Experimental and Modelled results

**Figure 2:**
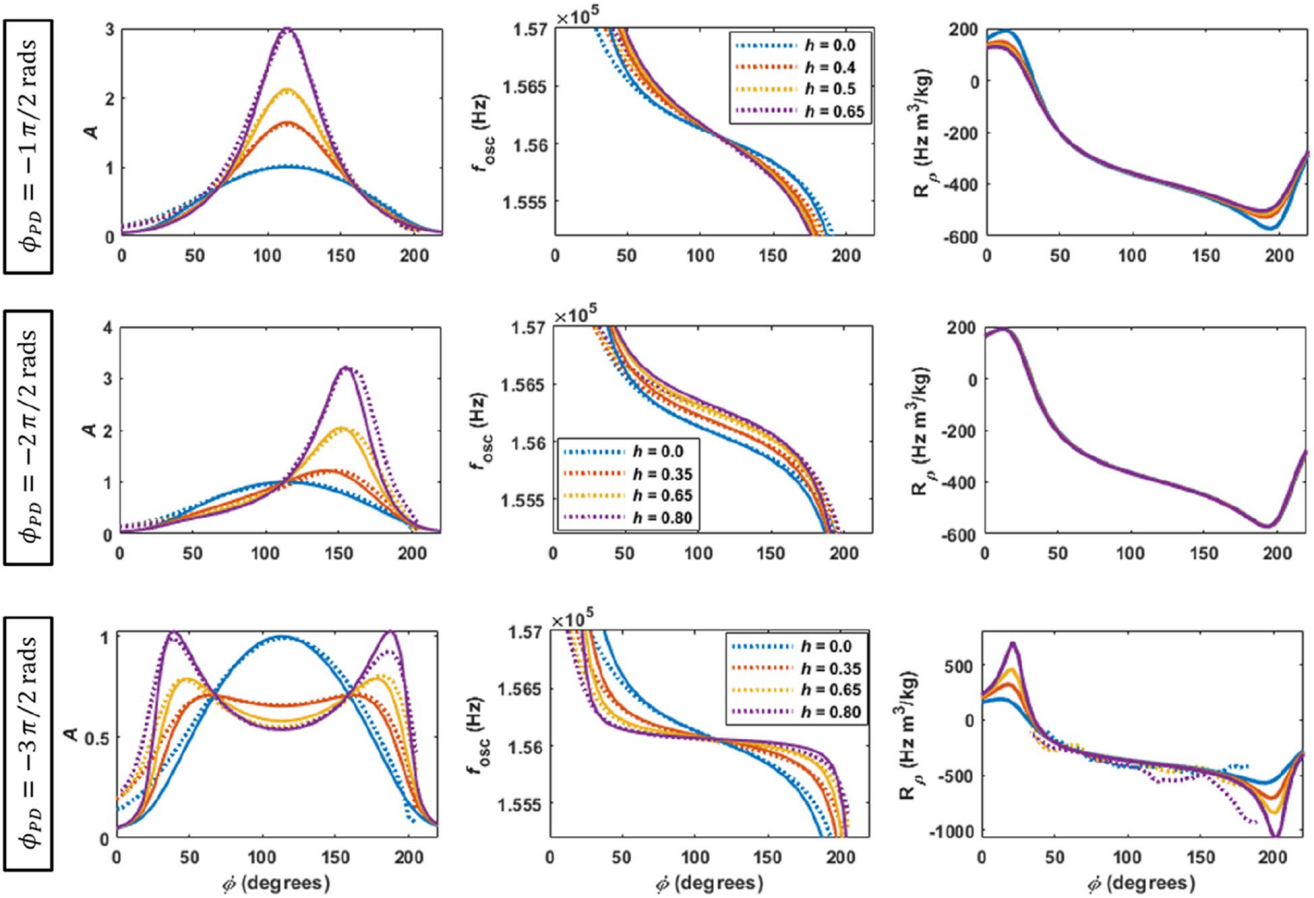
Modelled (solid lines) and experimental (dotted lines) amplitude and frequency of oscillation, and responsivities to density variations (3%), as function of the imposed phase ($\phi$) in the PLL and the parametric pump gain (*h*) and phase (${\phi }_{PD}$), for the closed cell filled with air. A constant transduction gain of 1/1100 is used to fit the experimental values of amplitude of oscillation to the modelled results.

The solid lines in Fig. 2 show the results obtained using Eqs. (1), (2) and (4) for the adimensional amplitude, frequency of oscillation and responsivity to density variations, as function of the phase imposed in the PLL ($\phi$) and the parametric pump gain (*h*) and phase (${\phi }_{PD}$). The dotted lines represent the experimental results.

The top row of Fig. 2 presents the case of ${\phi }_{PD}=-1\pi /2 \mathrm{rad}$, typical of parametric amplification^24^. It is observed that increasing the pump causes an increase in the amplitude of oscillation and quality factor around the resonance frequency, and also an increase of the slope of the phase response (which corresponds to the typical reduced slope in the $f$ vs ${\varphi }_{CT}$ plot)^2^. The modelled responsivity to density variations slightly decreases with the pump for imposed phases $\phi$ away from the resonance.

The middle row shows the case of ${\phi }_{PD}=-2\pi /2 \mathrm{rad}$, in which the amplitude of oscillation increases with the parametric pump in the region of higher imposed phases $\phi$^21^, while the oscillation frequency response translate, with constant slope, to higher frequencies (as if the spring constant of the microcantilever increased)^3^. No dependence of the responsivity to the density variations with the parametric pump is observed.

The bottom row presents the case of ${\phi }_{PD}=-3\pi /2 \mathrm{rad}$, typical of parametric supression^20^. This case is characterised by a symmetrical growth of the amplitude of oscillation away from the resonance and a suppression of the amplitude around the resonance frequency. The slope of the phase response reduces with the parametric pump^20^ (or increases in a typical $f$ vs ${\varphi }_{CT}$ plot^2^). The regions of increased amplitude correspond to regions of increased responsivities, showing that, by appropriately choosing the imposed phase $\phi$ and the parametric pump gain, one can increase the responsivity. The region of increased responsivity to density was experimentally studied by measuring the oscillation frequency response at two values of pressure differing by $\sim 3\%$, and for different values of parametric pump. Then, the responsivity to density variations is calculated from the pressure difference, using Eq. (4) in the form of ${R}_{\rho }= \frac{\partial f}{\partial \rho }=\frac{\partial f}{\partial P}\frac{\partial P}{\partial \rho }=\frac{\partial f}{\partial P}\frac{RT}{MM}=\frac{0.08314 T}{MM}\left(\frac{{f}_{2}-{f}_{1}}{{P}_{2}-{P}_{1}}\right)$, with $MM=28.9$ g/mol the molar mass of air and $T=300 K$ the absolute temperature. Despite the noisier response of the experimental responsivity curves, the trends mentioned above can be observed. Details on the geometry of the cantilever used in experiments and simulations and on the pressure control setup can be found in “Materials and methods” section.

Figure 2 shows that the modelled and experimental dynamical responses are in good agreement in the central region of the curves, which correspond to regions with high amplitude of oscillation. However, on the edges of the curves (corresponding to regions of low amplitudes), the analytical model disagrees with the experimental response. This is attributed to the loss of effectiveness of the PI controller due to the low amplitude of deflection. In fact, for small amplitudes, the error signal *X* becomes small (see Eq. (S15) in Supplementary Materials), and, consequently, the PI controller becomes slow and the steady-state condition (*X* = 0), which is the basic assumption of the analytical model, is not well satisfied.

Note that the case of ${\phi }_{PD}=-0\pi /2 \mathrm{rad}$ was not plotted. This is similar to the case of ${\phi }_{PD}=-2\pi /2 \mathrm{rad}$, except that the amplitude increases at small imposed phases and the frequency oscillation translate to lower values with constant slope. The responsivity to density variations also remains unchanged with parametric pumping. The four cases of ${\phi }_{PD}$ discussed here transition smoothly into each other when using intermediate values of ${\phi }_{PD}$, with a periodicity of $2\pi$.

### Real-time frequency shifts and frequency stability

The limit of detection of density changes is defined by ${LoD}_{\rho }=3\frac{\delta {f}_{min}}{{R}_{\rho }}$, where $\delta {f}_{min}$ is the minimum detectable frequency shift in the system, ${R}_{\rho }$ is the responsivity to density changes as defined in Eq. (4), and a 3-standard deviation of frequency change is understood as a measurement instead of noise.

Therefore, ${LoD}_{\rho }$ depends on the frequency stability of the system^26,27^, which is quantified in the time domain by the Allan variation

5 $$\sigma_{y}^{2} \left( \tau \right) = \frac{1}{{2\left( {M - 1} \right)}}\mathop \sum \limits_{i = 1}^{M - 1} \left( {f_{i + 1} - f_{i} } \right)^{2} ,$$

with *M* is the total number of frequency measurements and ${f}_{i}$ the *i*th frequency measurement (averaged in the time window with duration $\tau$).

**Figure 3:**
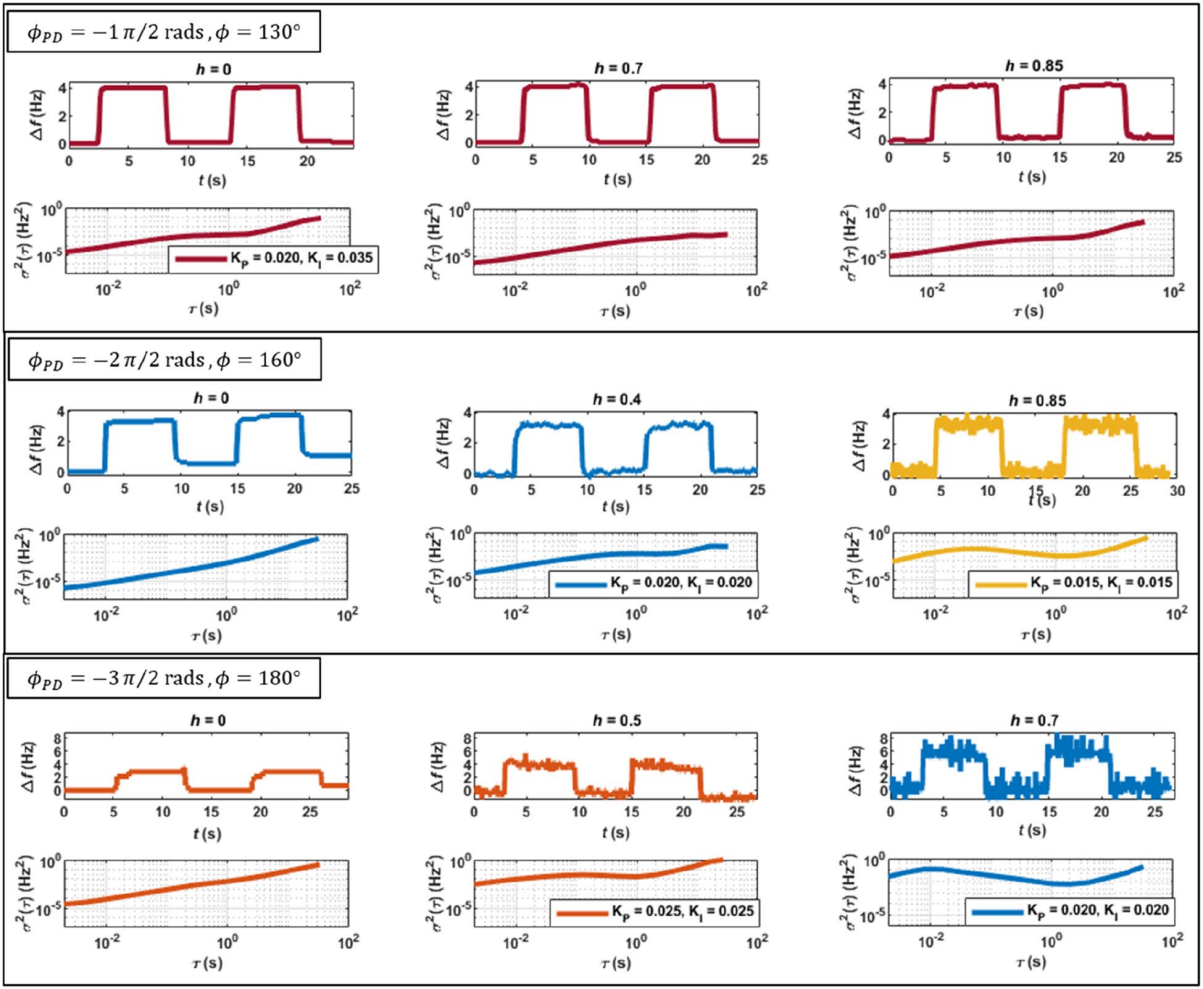
Real-time shifts of frequency caused by pressure cycles (7 mbar) in the closed cell and Allan variation of the resonator, as function of the parametric pump gain *h* and phase ${\phi }_{PD}$, for fixed imposed phases $\phi$ in the PLL. Each pair of controller conditions (*K*_P_ and *K*_I_) is represented by a different color.

Figure 3 shows real-time frequency shifts induced by pressure cycles of 7 mbar and the Allan variation of the system (frequency values collected at 1000 sample/s for 100 s), as functions of the parametric pump gain (*h*) and phase (${\phi }_{PD})$, considering fixed imposed phases ($\phi$) in the PLL. The top row shows the case of ${\phi }_{PD}=-1\pi /2 \mathrm{rad}$ and $\phi =130^\circ$. It is observed that the pressure-induced frequency shifts of $\sim 4 \mathrm{Hz}$ are independent on the parametric gain, as discussed in Fig. 2. In addition, the frequency response is smooth even for high parametric pump gains, which is confirmed by the low (and also independent on the pump level) Allan variation curves at small integration times.

In the case of ${\phi }_{PD}=-2\pi /2 \mathrm{rad}$ and $\phi =160^\circ$, shown in the middle row, the same $\sim 4 \mathrm{Hz}$ pressure-induced frequency shifts are also independent on parametric pump gain, as previously discussed in Fig. 2. However, it is evident that the frequency noise increases progressively with the parametric pump. This is also confirmed by the Allan variation curves, which increase of 2–3 orders of magnitude with the parametric pump in the low integration times.

Finally, the case of ${\phi }_{PD}=-3\pi /2 \mathrm{rad}$ and $\phi =180^\circ$ is shown in the bottom row. The improved responsivity to density changes with the parametric pump (expected from Fig. 2), is indeed confirmed, since the initial pressure-induced shifts of $\sim 4 \mathrm{Hz}$ almost double to $\sim 7 \mathrm{Hz}$ at high pump gains. However, a substantially increase in frequency noise is also observed in the frequency curves, also confirmed by the 2–3 orders of magnitude increase in the low integration times of the Allan variation curves. The increase of responsivity is accompanied by an even bigger increase of noise in the system, worsening the limit of detection.

The experiments leading to the results presented in Fig. 3, suggest that the frequency noise in the PLL increases with the parametric gain for all chosen ${\phi }_{PD}$, except for the case of ${\phi }_{PD}=-1\pi /2 \mathrm{rad}$. Similar results have been recently reported by other authors for the case of parametric pumped resonators in open-loop configuration^2,19^.

A more complete characterization of the Allan variation curves and the distribution of the values of *X* and *Y* components of the deflection in the closed-loop PLL configuration are shown in Figs. S2 and S6 of the Supplementary Material.

### Noise-driven fluctuations in *X* and *Y* signals in open-loop configuration

To clarify if the frequency noise observed in the PLL (measured with the Allan variation, see also Fig. S2 and S6 in the Supplementary Materials) is due to the parametrically pumped resonator dynamics or, instead, to the closed-loop PLL dynamics, the loop was opened and the noise-driven fluctuations in *X* and *Y* signals measured.

**Figure 4:**
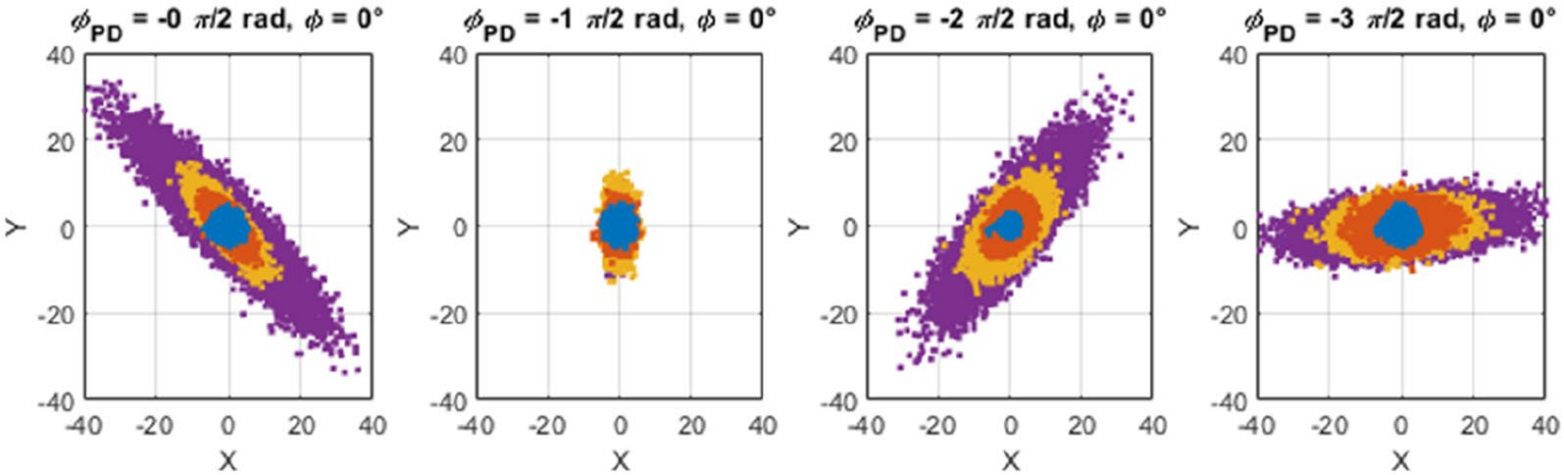
Experimental distribution of *X* and *Y* values as function of the parametric phase $({\phi }_{PD})$ and gain (*h* from from 0 to ~ 0.8, blue-yellow-orange-purple), for noise-driven deflection in open-loop configuration.

This experiment consisted of disconnecting the direct force (with the PI-controller) to the piezo, while still using the DDS-dither to synthetize the modulation signal, this time at precisely $2{\omega }_{0}$. This signal is amplified by the linear amplifier, phase shifted by ${\phi }_{PD}$, multiplied by the noise-driven deflection, and fed to the piezo. Simultaneously, the DDS-sine and DDS-cosine create the reference signals, at ${\omega }_{0}$ and $\phi =0^\circ$, used to demodulate the noise-driven deflection and generate fluctuating *X* and *Y* signals that are measured for 60 s at 500 sample/s. The schematic describing this setup is shown in Fig. S3 of the Supplementary Material. The results are shown in Fig. 4, for different ${\phi }_{PD}$ and different parametric pump gains *h*. It can be observed that with no parametric pump (blue regions) the distribution of values is independent on the phase ${\phi }_{PD}$ and symmetric around the origin. When the parametric pump gain increases (orange-yellow-purple), the noise distribution stretches in one direction for all ${\phi }_{PD}$ expect for the case of ${\phi }_{PD}=-1\pi /2 \mathrm{rad}$.

These results show the phenomenon of mechanical noise squeezing^28^, and indicate that the frequency noise observed in the closed-loop PLL and discussed in Fig. 3 has its origin in the dynamics of the parametrically pumped resonator. In the next section, the fluctuation of *X*-signal measured in open-loop is modelled and used to explain the frequency noise observed in the closed-loop PLL.

### Power Spectral Density of the *X*-signal in open-loop and frequency noise in the closed-loop PLL

**Figure 5:**
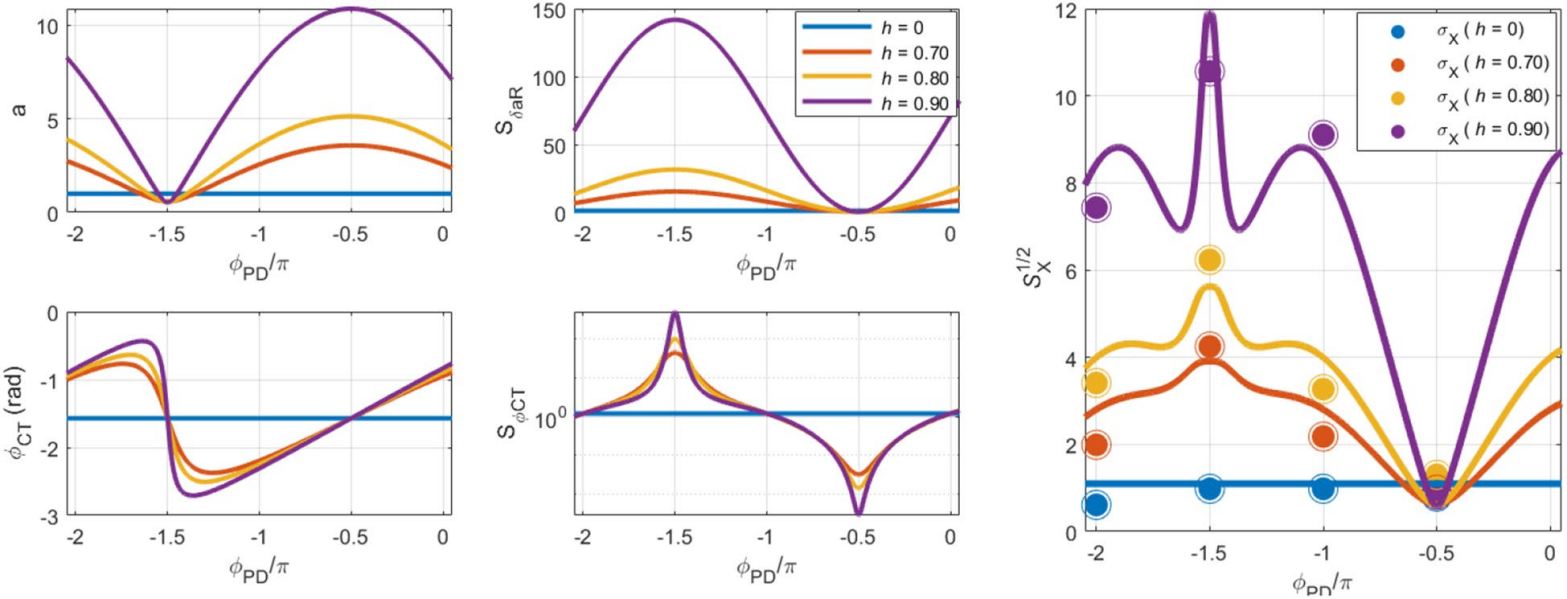
Left: Adimensional amplitude and phase of a parametrically pumped microresonator in open-loop; Center: PSD of the real part of the amplitude noise and phase noise in open-loop; Right: PSD of the *X* value in open-loop, calculated with Eq. (8), as function of the parametric gain and phase ${\phi }_{PD}$. The standard deviation of the *X* values measured experimentally in open-loop (see Fig. S5 of the Supplementary Material) are added to the PSD modelled curves.

The fluctuations in the *X*-signal (measured and modelled in open-loop—Figs. 4 and 5) can be readily translated to the frequency noise observed in the closed-loop PLL (discussed in Fig. 3) by assuming that a single value of the error component *X* in the PLL determines a univocal output value of frequency from the PI-controller. Both the open-loop and closed-loop PLL configurations show a similar noise response of the component *X,* as can be confirmed in Figs. S5 and S6 in Supplementary Material. As seen in the figures, the distribution of the *X* values are independent on the parametric pumping gain for the case of ${\phi }_{PD}=-1\pi /2 \mathrm{rad}$, and otherwise for the other considered phase cases.

The noise-driven deflection of the resonator in open-loop at ${\omega }_{0}$ can be divided into quadrature phases^3,28^ (see Fig. S3 in Supplementary Materials)

6 $$A\left( t \right) {\text{sin}}\left( {\omega_{0} t + \varphi_{CT} \left( t \right)} \right) = X_{1} \left( t \right){\text{ cos}}\left( {\omega_{0} t} \right) + X_{2} \left( t \right){\text{ sin}}\left( {\omega_{0} t} \right)$$

where ${X}_{1}\left(t\right)$ and ${X}_{2}\left(t\right)$ are random variables that vary slowly compared to ${\omega }_{0}$. After demodulation with the reference signals (with $\phi =0$, for simplicity) the *X*-signal is obtained as

7 $$X\left( t \right) = X_{1} \left( t \right) = A\left( t \right) {\text{sin}}\left( {\varphi_{CT} \left( t \right)} \right).$$

Applying propagation of error leads to the power spectral density (PSD) of the *X*-signal fluctuations

8 $$S_{X} = \left( {\frac{\partial X}{{\partial {\text{A}}}}} \right)^{2} S_{{\delta a_{R} }} + \left( {\frac{\partial X}{{\partial \varphi_{CT} }}} \right)^{2} S_{{\varphi_{CT} }} ,$$

where it is considered that ${S}_{X}={\sigma }_{X}^{2}$ is the variance of the *X* values and ${S}_{\mathrm{X}}^{1/2}={\sigma }_{X}$ its standard deviation. Equation (8) shows that the *X*-signal random fluctuation depends on the PSDs of the real part of the amplitude noise (${S}_{{\delta a}_{R}}$) and the phase noise of the cantilever resonator (${S}_{{\varphi }_{CT}}$).

The amplitude and phase noise of the parametrically pumped resonator in open-loop configuration have been analytically modelled in the work of Mohammadi et al^2^ (see the Supplementary Material for details). In this model, the amplitude noise is assumed to have uncorrelated real and imaginary parts, whose PSDs at ${\omega }_{0}$ are given by

9 $$S_{{\delta a_{R} }} = I_{th} \frac{{1 + \left| h \right|^{2} + 2\left| h \right| {\text{cos}}\left( {\phi_{PD} } \right)}}{{\left( {1 - \left| h \right|^{2} } \right)^{2} }}$$

10 $$S_{{\delta a_{I} }} = I_{th} \frac{{1 + \left| h \right|^{2} - 2\left| h \right| {\text{cos}}\left( {\phi_{PD} } \right)}}{{\left( {1 - \left| h \right|^{2} } \right)^{2} }},$$

with ${S}_{{\delta a}_{R}}$ and ${S}_{{\delta a}_{I}}$ the PSD of the real and imaginary parts of the amplitude noise, respectively, ${I}_{th}={\sigma }_{\Xi }^{2}=\frac{{Q}^{2}{\sigma }_{\mathrm{F}}^{2}}{2{m}_{0}^{2}{\omega }_{0}^{3}{x}_{c}^{2}}$ and ${\sigma }_{\mathrm{F}}^{2}=4{k}_{B}T\Gamma =\frac{4{k}_{B}T{m}_{0}{\omega }_{0}}{Q}$ the PSDs of the thermomechanical noise and thermomechanical force^3^, respectively, and $\left|h\right|=\frac{H{Q}_{R}}{2k}$.

Both components of the amplitude noise will contribute to the phase noise of the resonator. The PSD of the phase noise (${S\varphi }_{CT}$) is finally given by the expression

11 $$S\varphi_{CT} = S_{{\delta a_{R} }} \left( {\frac{{\left( {1 - \left| h \right| {\text{cos}}\left( {\phi_{PD} } \right)} \right)\left( {1 - \left| h \right|^{2} } \right)}}{{1 + \left| h \right|^{2} - 2\left| h \right| {\text{cos}}\left( {\phi_{PD} } \right)}}} \right)^{2} + S_{{\delta a_{I} }} \left( {\frac{{\left( { - \left| h \right| {\text{sin}}\left( {\phi_{PD} } \right)} \right)\left( {1 - \left| h \right|^{2} } \right)}}{{1 + \left| h \right|^{2} - 2\left| h \right| {\text{cos}}\left( {\phi_{PD} } \right)}}} \right)^{2} .$$

Equations (9) and (11) are finally used into Eq. (8) to determine the PSD of the fluctuations of the *X*-signal in open-loop configuration.

The left panels in Fig. 5 shows the adimensional amplitude and phase of the parametrically pumped microcantilever in the degenerate case, as function of the parametric gain *h* and phase ${\phi }_{PD}$ (see Eq. (S7) in the Supplementary Materials for details on the transfer function). The cases of parametric amplification and suppression can be readily found for ${\phi }_{PD}=-1\pi /2 \mathrm{rad}$ and ${\phi }_{PD}=-3\pi /2 \mathrm{rad}$, respectively. The middle panels plot the PSDs of the real part of the amplitude noise and the phase noise, as given by Eqs. (9) and 11.

Finally, the right panel shows the plot of the square root of Eq. (8), or the standard deviation of the modelled *X* values (${S}_{X}^{1/2}={\sigma }_{X}$). It can be seen that the standard deviation shows a minimum value for ${\phi }_{PD}=-1\pi /2 \mathrm{rad}$, mostly independent on the parametric gain *h*. For other values of ${\phi }_{PD}$, the standard deviation is larger and dependent on the parametric gain, with a maximum on ${\phi }_{PD}=-3\pi /2 \mathrm{rad}$. The standard deviation of the *X* values measured in open-loop configuration (presented in Fig. S5 of the Supplementary Material) are overlapped with the modelled results, showing a good agreement with the model proposed in Eq. (8).

## Discussion

Parametric pumping seems not useful in sensing applications requiring detecting frequency shifts in a closed-loop PLL configuration. The reason is the general increase of frequency noise that prevents detecting small shifts of frequency and, therefore, hinders the attainable limits of detection. Similar noise results in open-loop configuration have been reported recently^2,19^. Filtering the high frequency noise in the PLL can make it possible to recover the increased responsivity in the case ${\phi }_{PD}=-3\pi /2 \mathrm{rad}$, but at the expense of only detecting slow phenomena.

However, we conjecture that parametric pump in a PLL can still be useful if used to increase the robustness of control strategies^29–31^ or to optimise transient times. When performing the experiments leading to Fig. 3, it was observed that the PI-controller tuning seemed independent on the level of parametric pump when ${\phi }_{PD}=-1\pi /2 \mathrm{rad}$ (all the results presented in the top row of Fig. 3 use the same controller conditions, see also Fig. S2 in Supplementary Materials). The same was not true when other ${\phi }_{PD}$ were used. In fact, for ${\phi }_{PD}=-3\pi /2 \mathrm{rad}$, for example, it was extremely difficult to find proper working conditions for the PI-controller, and these needed to be readjusted often when the parametric pump changed. As discussed in Supplementary Material (see Eq. (S15)), the transient response of the PI-controller depends on the updated value of the error parameter $X$. This, in turn, depends on the amplitude and phase of the oscillation (see Eq. (7)). In the case of ${\phi }_{PD}=-1\pi /2 \mathrm{rad}$, there is a simultaneous increase of the amplitude of oscillation and a decrease of the slope of the phase response with the parametric pump gain, as discussed in Fig. 2. These two effects compensate each other, making it possible to keep the controller tuned with the same gains ${K}_{P}$ and ${K}_{I}$. This conjecture is based on observations resulting from the experiments presented in this work, and will require in-depth future work and dedicated experiments to assess its validity.

## Conclusions

In this work we presented a new topology of a PLL platform with a digital and PI-controlled parametrically pumped microcantilever. We extend existing linear models for the parametrically pumped resonator in open-loop, and incorporate them in the working conditions of the closed-loop PLL, considering, in addition, an explicit dependence on the properties of the viscous medium. This approach makes it possible to assess the responsivity of the system to density variations. The models for the dynamical response and the responsivity of the system are validated on the basis of experimental results. Conditions for improved responsivity of the system to density variations are found, but it is also shown that the frequency noise of the PLL increases with the parametric pump, restricting the attainable limit of detection. The frequency noise in the closed-loop PLL is studied and modelled by using existing analytical formulations for the amplitude and phase noise of a parametrically pumped resonator in open-loop configuration. We conclude that parametric pump in a PLL does not improve the sensing performance in applications requiring detecting frequency shifts.

## Materials and methods

### Microcantilever resonator

A doped single crystal silicon cantilever ACST-TL from AppNano with nominal dimensions of *L* = 130 µm, *W* = 29 µm, *T* = 2.7 µm was used in the experiments and simulations. A resonance frequency and quality factor in air of *f* = 156.045 kHz and *Q* = 300 were measured with a R9 SPM Controller from RHK Technology.

### Pressure characterisation

The closed cell is connected to a small PEEK container adapted with a rubber membrane through a polypropylene tubing system. This system is filled with air. The pressure is read with a sensor connected to the closed cell and is periodically cycled by forcing the rubber membrane with a weight.

### Control electronics

A bespoke board was developed with the electronics required to realize the control diagram shown in Fig. 1. It contains 3 Direct Digital Synthesizers (AD9833, Analog Devices Inc) sharing a common clock (24 MHz) and thus synchronized to generate references and dither-piezo signals. The *X*/*Y* demodulators are implemented with the analog processor AD630 by Analog Devices Inc. To avoid any aliasing problem, both the *X* and *Y* signals are filtered with a low-pass filter with a cut-off at 5 kHz. These signals are subsequently converted to digital with two 16-bit A/D converters (sampling rate > 10 kHz) and *X* used as input to the Microcontroller (Microchip dsPIC33EP512GP806), whose firmware contains the digital control loops algorithms. A Raspberry Pi is connected to the dsPIC microcontroller and to an external PC via a LAN cable, sending commands to the microcontroller, transmitting data and reading the frequency, *X* and *Y* values in real time.

### PLL dynamics and performance

To achieve high-resolution frequency generation, the DDSs contain phase accumulators with 28 bits and the clock frequency is set at 24 MHz. With these parameters, the theoretical frequency resolution is 24 × 10^6^/2^28^ = 0.0894 Hz. Setting the driving clock at 24 MHz allows to get very accurate digitized waveforms in the hundred-of-kHz region. The range of the attainable lock-in frequency in the DDSs can be selected from 5.86 kHz up to several hundreds of kHz. These different frequency ranges are divided into 2^16^ frequency steps that are used by the digital PI-controller to increment or decrement the driving signal following any variation of the resonator dynamics. In applications requiring detecting tiny frequency shifts, it is appropriate to choose the lock-in range so that the minimum frequency step stays below the frequency noise from any noise sources. Assuming a frequency noise equal to 2 times the minimum frequency step, a dynamic range (i.e. range/noise) of 2^15^ will result (90 dB). However, other experimental factors can further limit the attainable dynamic range of the PLL. As explained, the PI-controller requires some amplitude of oscillation to reach the steady-state. In case the resonance peak has a low quality factor, for example, it becomes large and the PLL will not be able to lock-in in all the frequencies of the peak due to the low amplitude in the extremities of the curve. This can be seen, for example, in the experimental plots in Fig. 2 (dotted lines). Here, considering the case of parametric amplification (top row), the PI-controller responds well in the central region of the curves (due to the high amplitude of oscillation), in region of 155.5 kHz to 156.5 kHz (1000 Hz). This region can be covered with a frequency step of the mentioned 0.0894 Hz (assuming the noise floor is below this frequency step), corresponding to a dynamic range of ~ 80 dB.

The possibility of controlling the imposed phase between the direct forcing and the reference demodulating signals has proved interesting for sensing applications, since, as shown, it is not always preferred to work at resonance ($\pi /2$ rad), and some different phases may be more advantageous^25^.

### Electronic circuit for implementing parametric pumping

The generation of the signal at 2*ω* is achieved by using a multiplier AD835 (Analog Devices Inc.), which multiplies the DDS-dither synthetized signal by a $\pi /2$ rad shifted version of itself. The multiplication of the signal at 2*ω* by the deflection of the cantilever is performed by the analog multiplier AD633 (Analog Devices Inc.). The Phase-Shifter used to control ${\phi }_{PD}$ is described in detail in^32^. It consists of two stages in series, each one working as an all-pass filter and able to shift the phase of the signal by at most 180°, by individually operating two adjustable potentiometers.

The modulation of the spring constant consists, in fact, of modulating the direct forcing term in real-time, with the same effect. Similar strategies were previously described in references^14,15^ for inducing parametric resonance and in^21^ for parametric pumping in a feedback loop. The fact that the signal from the 4-quadrant detector is not differentiated or integrated makes the PLL extremely fast, and only a minimal delay is introduced between detection of the deflection and the parametric pump modulation. In addition, there is no need of an external apparatus to induce the parametric pump and the direct force and parametric pump signals are synthetized with very low noise and triggered.

## Supplementary Information

Supplementary Information.

## Data Availability

The datasets generated during and/or analysed during the current study are available from the corresponding author on reasonable request.
