# Supplementary material for: Dynamical response and noise limit of a parametrically pumped microcantilever sensor in a Phase-Locked Loop
Source: Sci Rep. 2023 Feb 7;13:2157. doi: 10.1038/s41598-023-29420-3 (PMC9905076; doi:10.1038/s41598-023-29420-3)
Supplement: Supplementary file 1 — Supplementary Information. [file 41598_2023_29420_MOESM1_ESM.docx]

**Dynamical response and noise limit of a parametrically pumped microcantilever sensor in a Phase-Locked Loop**

**João Mouro, Paolo Paoletti, Marco Sartore and Bruno Tiribilli**

**Part I. Frequency and Amplitude of oscillation of a parametrically pumped cantilever, subject to hydrodynamic load, in the Phase-Locked Loop**

Fig. S1 sketches the propagation of the electrical signals around the implemented Phase-Locked Loop and will be used to illustrate the derivation of the analytical model.


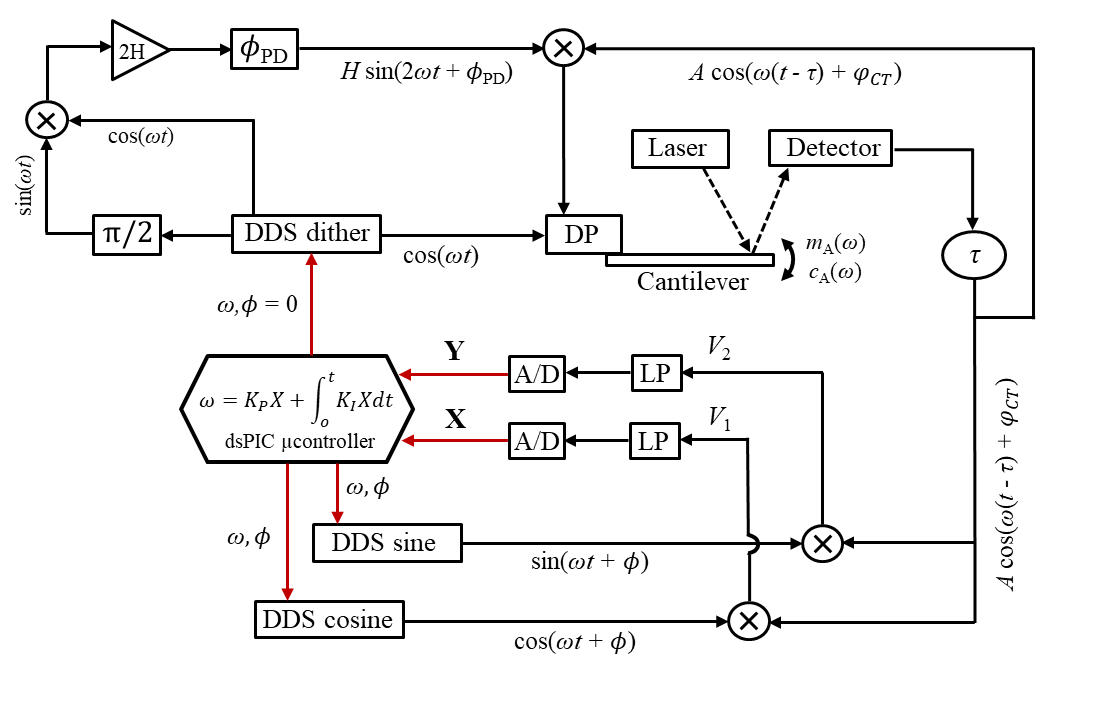


**Figure S1** Schematic of the electrical signals throughout the developed PLL platform.

**I.1. Frequency and amplitude of oscillation in the Phase-Locked Loop**

I.1.1. Frequency of oscillation

The output of the multiplication of the deflection signal by the demodulating cosine reference is given by

$V_{1}=A\cos\left( \omega t-\omega\tau+\varphi_{CT} \right)\cos\left( \omega t + \phi\right)$

where $A$ and $\omega$ are the amplitude and frequency of the cantilever deflection, $\varphi_{CT}$ is the phase of the cantilever response with respect to the excitation force, $\phi$ is the imposed phase in the system and $\tau$ is the total delay of the signals around the feedback loop (mostly due to the propagation of the acoustic waves through the holder of the cantilever).

The low-pass filter removes all high-frequency terms and the DC signal for the in-phase error parameter *X* is obtained

$X=\frac{A}{2}\left( \cos\theta\cos\phi+\sin\theta\sin\phi\right)=\frac{A}{2}\cos\left( \theta-\phi\right)$ (S1)

Where $\theta=-\omega\tau+\varphi_{CT}$ was defined.

Assuming a well-tuned controller, the steady-state system will have *X =* 0, implying the phase condition

$\cos\left( \theta-\phi\right)=0 \underset{\Rightarrow}{} -\omega\tau+\varphi_{CT}-\phi=-\left( \frac{\pi}{2}+n\pi\right)$, with $n=0, 1 ,2, \ldots$ (S2)

The frequency of oscillation can be calculated from this phase condition, after substituting $\varphi_{CT}$ by the expression for the phase of a parametrically pumped resonator, as discussed next.

I.1.2. Amplitude of oscillation

The output of the multiplication of the deflection signal by the demodulating sine reference is given by

$V_{2}=A\cos\left( \omega t+\theta\right)\sin\left( \omega t + \phi\right)$

Similarly, after removing the high-frequency terms with the filter, the DC signal for the quadrature parameter *Y* is obtained

$Y=\frac{A}{2}\left( \cos\theta\sin\phi-\sin\theta\cos\phi\right)=\frac{A}{2}\sin\left( \phi-\theta\right)=\frac{A}{2}\sin\left( -\beta\right)$ (S3)

Where $\beta=-\phi+\theta$ is defined. The frequency condition *X* = 0 of equation (S2) implies that

$\cos\left( \theta-\phi\right)=0 \underset{\Rightarrow}{}$ $\cos\left( \beta\right)=0 \underset{\Rightarrow}{}$ $\beta=-\left( \frac{\pi}{2}+n\pi\right)$, with *n* = 0, 1, 2, …

And, therefore, *Y* is given by

$Y=\frac{A}{2}\sin\left( \frac{\pi}{2}+n\pi\right)=\left\{ \begin{aligned} \frac{A}{2}, for n = 0, 2, 4,\ldots\\ -\frac{A}{2}, for n = 1, 3, 5,\ldots\end{aligned} \right.$ (S4)

With *A* the amplitude of oscillation of a parametrically pumped cantilever.

**I.2. Transfer function of a parametrically pumped resonator in adimensional form**

The general equation of motion of the parametrically pumped linear harmonic oscillator is given by

$m\frac{d^{2}x}{{dt}^{2}}+\Gamma\frac{dx}{dt}+m\omega_{0}^{2}x+H\sin\left( \omega_{P}t+\phi_{PD} \right)x=G\cos\left( \omega_{D}t \right)$ (S5)

Where $m$ is the effective mass, $\omega_{0}$ is the natural frequency of the resonator, $\Gamma$ the linear damping rate, $G$ is the harmonic driving force at a frequency $\omega_{D}$, and $\omega_{P}$ is the frequency of the parametric pump used to modulate the elastic constant with a gain $H$ at a phase $\phi_{PD}$ with respect to the drive force.

This equation of motion can be adimensionalised, as in Lifshitz^1^ and Mohammadi^2^, to obtain

$\frac{d^{2}\tilde{x}}{{d\tilde{t}}^{2}}+\frac{1}{Q}\frac{d\tilde{x}}{d\tilde{t}}+\left( 1+2\frac{\left| h \right|}{Q}\sin\left( \left( 2+\frac{\Omega_{P}}{Q} \right)t+\phi_{PD} \right) \right)\tilde{x}=\frac{\left| g \right|}{Q^{3/2}}\cos\left( \left( 1+\frac{\Omega_{D}}{Q} \right)t \right)$ (S6)

where $\tilde{x}=x/x_{c}$ and $\tilde{t}=\omega_{0}t$ are the adimensional amplitude of deflection and time, respectively, with $x_{c}$ the critical threshold of nonlinearity. $Q^{-1}=\frac{\Gamma}{m\omega_{0}}$is the quality factor, $\Omega_{D}=Q\left( \frac{\omega_{D}-\omega_{0}}{\omega_{0}} \right)$ and $\Omega_{P}=Q\left( \frac{\omega_{P}-2\omega_{0}}{\omega_{0}} \right)$ are the adimensional drive and parametric pumping frequencies, $\left| g \right|=\frac{Q^{3/2}G}{m\omega_{0}^{2}x_{c}}$ is the adimensional direct forcing term, and $\left| h \right|=\frac{HQ}{2k}$ is the adimensional strength of the parametric pump, with $k$ its spring constant. $H_{th}=\frac{2k}{Q}$ is defined as the threshold value that causes the system to reach instability and enter in parametric resonance regime^3^.

In the present work, the resonator is kept below the threshold of parametrically resonance and only the degenerate case is used. Therefore, the pumping frequency is exactly twice the drive frequency, $\omega_{P}=2\omega_{D}$, or, in adimensional form $\Omega_{P}=2\Omega_{D}$.

Equation (S6) can be solved by secular perturbation theory to obtain the transfer function of the parametrically pumped cantilever, given by^1,2^

$a= -\left| g \right|\left[ \frac{2\Omega_{D}+\left( i+\left| h \right|e^{-i\phi_{PD}} \right)}{4\Omega_{D}^{2}+1-\left| h \right|^{2}} \right]$ (S7)

The phase and amplitude of the parametrically pumped cantilever in the degenerate case are obtained from the transfer function, as

$\varphi_{CT}= \mathrm{arctg}\left[ \frac{1-\left| h \right| \sin\left( \phi_{PD} \right)}{2\Omega_{D}+\left| h \right| \cos\left( \phi_{PD} \right)} \right]$ (S8)

$\left| \frac{a}{g} \right|= \frac{\left[ \left( 2\Omega_{D}+\left| h \right| \cos\left( \phi_{PD} \right) \right)^{2}+\left( 1-\left| h \right| \sin\left( \phi_{PD} \right) \right)^{2} \right]^{\frac{1}{2}}}{4\Omega_{D}^{2}+1-\left| h \right|^{2}}$ (S9)

**I.3. Transfer function of the parametrically pumped cantilever subject to the hydrodynamic load**

The dimensional equation of motion for the system analysed in this work is given by

$\left( m_{0}{+ m}_{A} \right)\ddot{x}+\left( \frac{\omega_{0}m_{0}}{Q_{0}}+c_{A} \right)\dot{x}+k\left[ 1-H\left( \sin(2\omega t+\phi_{PD} \right)) \right]x=G \cos(\omega(t-\tau)- \phi)$ (S10)

As indicated in Fig. S1, the degenerate case of $\omega_{P}=2\omega_{D}=2\omega$ is implemented by the circuit. In this equation, $m_{A}$ and $c_{A}$ are the added mass and damping due to the surrounding fluid, with $Q_{0}$ the quality factor of the resonator in vacuum.

Sader^4^ and Maali^5^ developed analytical expressions to describe the added mass and damping caused by the fluid, from which an explicit dependence on the density and viscosity of the medium can be obtained

$m_{A}=\frac{\pi}{4}\rho LW^{2}\left( a_{1}+\frac{a_{2}}{W}\sqrt{\frac{2\eta}{\rho\omega}} \right)$ (S11a)

$c_{A}=\frac{\pi}{4}\rho{LW}^{2}\omega\left( \frac{b_{1}}{W}\sqrt{\frac{2\eta}{\rho\omega}}+\frac{b_{2}}{W^{2}}\frac{2\eta}{\rho\omega} \right)$ (S11b)

In these equations, $\omega$ is the oscillation frequency of the resonator in the viscous medium, $\rho$ and $\eta$ are the density and viscosity of the fluid, *a*_1_ = 1.0553, *a*_2_ = 3.7997, *b*_1_ = 3.8018, and *b*_2_ = 2.7364 are constants to describe the hydrodynamic function and $L$ and $W$ are the length and width of the microcantilever.

The resonance frequency, $\omega_{R}$, and quality factor of the resonator, $Q_{R}$, immersed in viscous fluids are described by the expressions^4^

$\omega_{R}=\omega_{0}\left( 1+\frac{m_{A}}{m_{0}} \right)^{-\frac{1}{2}}$ (S12a)

$Q_{R}=\omega_{R}\left( \frac{m_{0}+m_{A}}{c_{0}+c_{A}} \right)$ (S12b)

**I.4. Frequency and amplitude of oscillation of a parametrically pumped cantilever in a PLL**

The phase condition given by of equation (S2) can be explicitly written for the parametrically pumped cantilever, in the degenerate case, by considering the cantilever phase given by equation (S8)

$-\omega\tau+\mathrm{arctg}\left[ \frac{1-\frac{HQ_{R}}{2k} \sin\left( \phi_{PD} \right)}{2Q_{R}\left( \frac{\omega-\omega_{R}}{\omega_{R}} \right)+\frac{HQ_{R}}{2k} \cos\left( \phi_{PD} \right)} \right]-\phi=-\left( \frac{\pi}{2}+n\pi\right)$, with $n=0, 1 ,2, \ldots$ (S13)

The parameter $n$ describes the branch where the cantilever response can be found and depends on $\tau$. Substituting $Q_{R}$ in equation (S13) by the expressions given by equations (S11a), (S11b), (S12a) and (S12b), allows to obtain the phase condition of the PLL with an explicit dependence on the rheological parameters of the fluid.

Equation (S13) is numerically solved for $\omega$, as a function of the imposed phase in the system $\phi$, the parametric gain $H$ and the phase between the parametric pump and the direct force term $\phi_{PD}$.

The (adimensional) amplitude of oscillation (and the *Y*-signal described by equation (S4)) can then be calculated by

$A= \frac{\left[ \left( 2Q_{R}\left( \frac{\omega-\omega_{R}}{\omega_{R}} \right)+\frac{HQ_{R}}{2k} \cos\left( \phi_{PD} \right) \right)^{2}+\left( 1-\frac{HQ_{R}}{2k} \sin\left( \phi_{PD} \right) \right)^{2} \right]^{\frac{1}{2}}}{{2Q_{R}\left( \frac{\omega-\omega_{R}}{\omega_{R}} \right)}^{2}+1-\left( \frac{HQ_{R}}{2k} \right)^{2}}$ (S14)

Where $Q_{R}$ and the frequency of oscillation calculated with equation (S13) must be used.

**I.5. Response of the PI-controller**

The frequency of oscillation $\omega$ is continuously set by the PI-controller implemented in the dsPIC microcontroller, by

$\omega_{i+1}=\omega_{i}+K_{P}X+\int_{o}^{t} K_{I}Xdt=\omega_{i}+K_{P}\frac{A}{2}\cos\left( \theta-\phi\right)+\int_{o}^{t} K_{I}\frac{A}{2}\cos\left( \theta-\phi\right)dt$ (S15)

The updated value of the frequency, $\omega_{i+1}$, depends on the former value $\omega_{i}$, the value of $X$ (and consequently the amplitude and phase of the oscillation) and the gains $K_{P}$ and $K_{I}$.

This equation describes the transient response of the system, when some parameter of the medium changes. In steady-state, and provided the controller is properly tuned, the error $X$ is close to zero, and the frequency set by the controller is constant.

**Part II. Noise of the parametrically pumped microcantilever in closed-loop PLL**

Fig. S2 shows results of the Allan variation curves (left) and the distribution of the *X* and *Y* values (right), measured as function of the PI controller conditions and the parametric pumping phase ($\phi_{PD}$) and gain (*h)*, for fixed imposed phases in the PLL ($\phi$). As already partially shown in Fig. 3 of the manuscript, the Allan variation curves do not depend on the level of parametric pump in the case of $\phi_{PD}=-1\pi/2 \mathrm{rad}$. Conversely, in the cases of $\phi_{PD}=-2\pi/2 \mathrm{rad}$ and $\phi_{PD}=-3\pi/2 \mathrm{rad}$ it is possible to observe that the Allan variation curves increase several orders of magnitude with the parametric pumping gain in the small integration time region (sub-second regime).

**
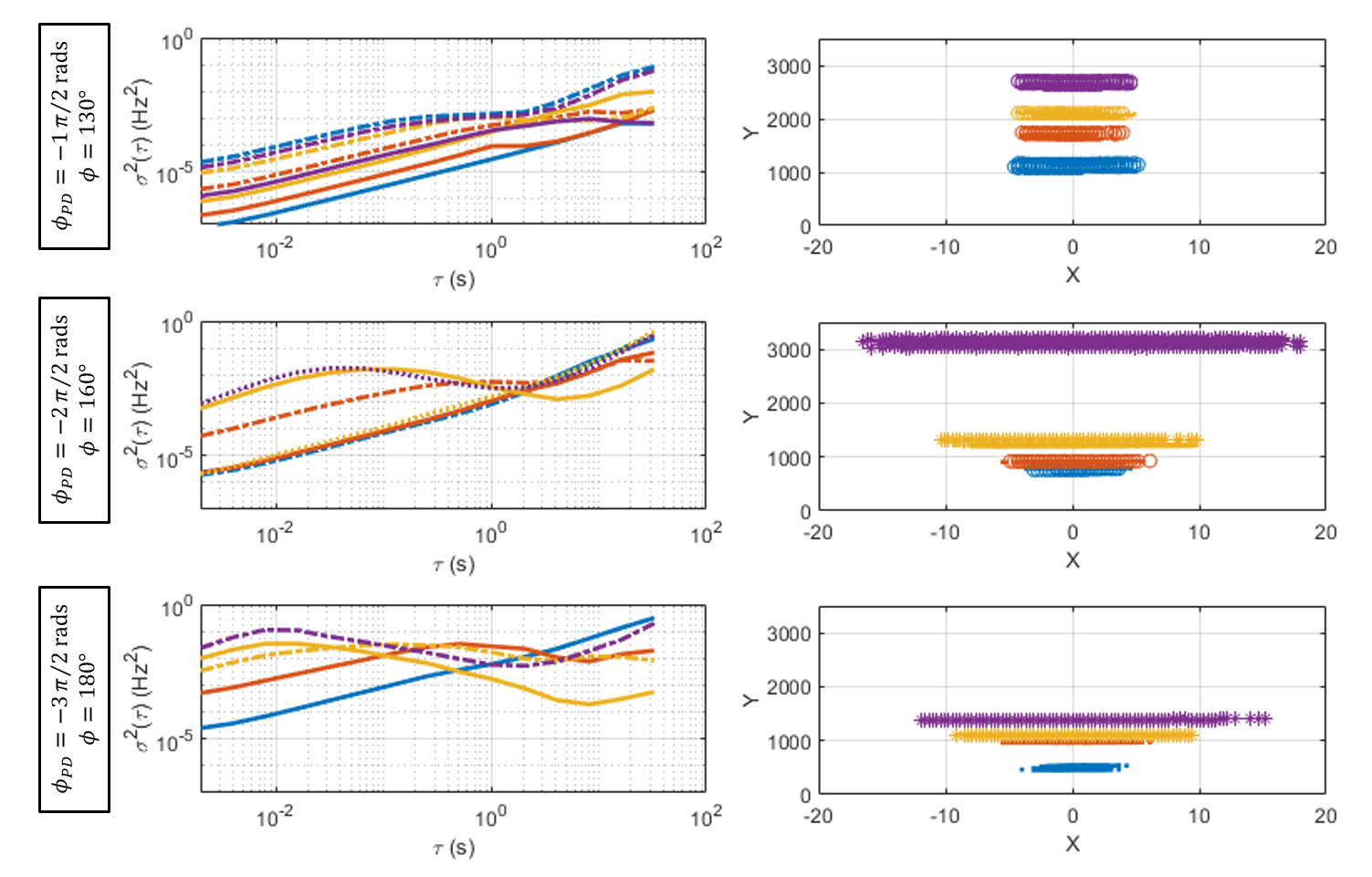
**

**Figure S2** Experimental Allan variation curves (left panels) and distribution of *X* and *Y* values (right panels), as function of the parametric phase ($\phi_{PD}$) and gain (*h* from 0 to ~0.80, blue-orange-yellow-purple curves), for fixed imposed phase in the PLL ($\phi$), measured in PLL closed-loop configuration. Three distinct PI-controller conditions are shown: *K*_P_ = 0.025, *K*_I_ = 0.025 (solid lines/ . symbols), *K*_P_ = 0.020, *K*_I_ = 0.035 (dotted lines/ o symbols) and *K*_P_ = 0.020, *K*_I_ = 0.020 (dashed-dotted lines/ * symbols).

Similarly, the distribution of the *X* values is also independent on the parametric pumping gain in the case of $\phi_{PD}=-1\pi/2 \mathrm{rad}$. In the other cases ($\phi_{PD}=-2\pi/2 \mathrm{rad}$ and $\phi_{PD}=-3\pi/2 \mathrm{rad}$), the distribution of the *X* values gets bigger with the parametric pumping level. Note that the values of *Y* follow the amplitudes of deflection shown in Fig. 2 of the manuscript, for each case.

**Part III. Noise of the parametrically pumped microcantilever in open-loop.**

Fig. S3 illustrates the setup used to obtain the random fluctuations of the values of *X* and *Y* and their dependence with the parametric phase $\phi_{PD}$ in open-loop.

**
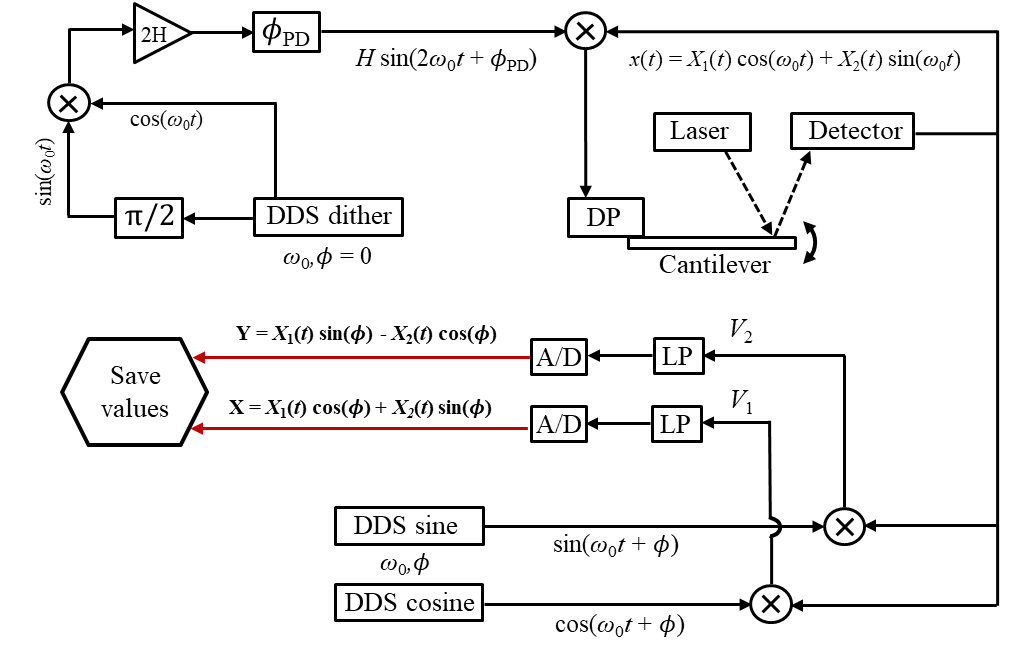
**

**Figure S3** Schematic of the electrical signals throughout the experimental setup used to measure the random variations of *X* and *Y* in an open-loop configuration.

The model developed here to calculate the Power Spectral Density (PSD) of the measured values of *X*Q follows closely the analytical model derived in Mohammadi et al^2^, which will be revisited here in detail, before extending it to explain our results.

**III.1. Amplitude, phase and frequency noise in a parametrically pumped resonator in open-loop**

III.1.1. Amplitude noise

The transfer function of the parametrically pumped resonator as presented in Mohammadi et al^2^ is

$\frac{a}{\left| g \right|}=\frac{-2\Omega_{D}-i+i\left| h \right|e^{i\phi_{PD}}}{4\Omega_{D}^{2}+1-\left| h \right|^{2}}= \frac{-2\Omega_{D}-\left| h \right| \sin\left( \phi_{PD} \right)}{4\Omega_{D}^{2}+1-\left| h \right|^{2}}+i\frac{-1+\left| h \right| \cos\left( \phi_{PD} \right)}{4\Omega_{D}^{2}+1-\left| h \right|^{2}}=a_{R}+{ia}_{I}$ (S16)

In this work the authors define the parametric pump phase $\phi_{PD}$ corresponding to the ‘attenuation’ and ‘gain’ cases as $\phi_{PD}=0 (2\pi)$ and $\phi_{PD}=\pi$, respectively, as opposed to the angles defined in the transfer function given by equation (S7). The phase and adimensional amplitude of the parametrically pumped resonator are then given by

$\varphi_{CT}=arctg\left[ \frac{Im}{Re} \right]=\mathrm{arctg}\left[ \frac{a_{I}}{a_{R}} \right]=\mathrm{arctg}\left[ \frac{1-\left| h \right| \cos\left( \phi_{PD} \right)}{2\Omega_{D}+\left| h \right| \sin\left( \phi_{PD} \right)} \right]$ (S17)

$\left| \frac{a}{g} \right|=a=\sqrt{a_{R}^{2}+a_{I}^{2}}=\frac{\left[ 4\Omega_{D}^{2}+4\Omega_{D}\left| h \right| \sin\left( \phi_{PD} \right)+1+\left| h \right|^{2}-2\left| h \right| \cos\left( \phi_{PD} \right) \right]^{1/2}}{4\Omega_{D}^{2}+1-\left| h \right|^{2}}$ (S18)

The amplitude given by equation (S18) allows to define the Power Spectral Density (PSD) of the amplitude noise, admitting two uncorrelated noise components, in the form of $\delta a={\delta a}_{R}+i{\delta a}_{I}$. Using the properties

$\mathrm{var}\left( ax+by \right)=a^{2}\mathrm{var}\left( x \right) + b^{2}\mathrm{var}\left( y \right)+2ab \mathrm{cov}(x,y)$

$\mathrm{var}\left( x \right)=\mathrm{cov}\left( x,x \right)=\sigma_{x}^{2}=S_{x}$

and considering that ${\delta a}_{R}$ and ${\delta a}_{I}$ are uncorrelated, one gets^2^

$S_{{\delta a}_{R}}=I_{th}\frac{1+\left| h \right|^{2}+4\Omega_{D}^{2}+2\left| h \right| \cos\left( \phi_{PD} \right)+4\Omega_{D}\left| h \right| \sin\left( \phi_{PD} \right)}{\left( 1-\left| h \right|^{2}+4\Omega_{D}^{2} \right)^{2}}$ (S19)

$S_{{\delta a}_{I}}=I_{th}\frac{1+\left| h \right|^{2}+4\Omega_{D}^{2}-2\left| h \right| \cos\left( \phi_{PD} \right)-4\Omega_{D}\left| h \right| \sin\left( \phi_{PD} \right)}{\left( 1-\left| h \right|^{2}+4\Omega_{D}^{2} \right)^{2}}$ (S20)

where $I_{th}=\sigma_{\Xi}^{2}=\frac{Q^{2}\sigma_{F}^{2}}{2m_{0}^{2}\omega_{0}^{3}x_{c}^{2}}$ is the adimensional PSD of the thermomechanical noise^2^, given by $\Xi=\Xi_{R}+i\Xi_{I}$, where $\sigma_{F}^{2}$ is the PSD of the thermomechanical force^6^ given by the expression $\sigma_{F}^{2}=4k_{B}T\Gamma=\frac{4k_{B}Tm_{0}\omega_{0}}{Q}$. The magnitude of $I_{th}$ therefore only depends on the temperature, quality factor, mass and frequency of the resonator, but not on the parametric pump strength^2,6^.

III.1.2. Phase noise

It is assumed that the phase noise of the microresonator is a function of both the components of the amplitude noise, $\varphi_{CT}(a_{R},a_{I})$, such that

${\partial\varphi}_{CT}= \left( \frac{{\partial\varphi}_{CT}}{\partial a_{R}} \right) {\delta a}_{R}+\left( \frac{{\partial\varphi}_{CT}}{\partial a_{I}} \right) {\delta a}_{I}$ (S21)

And, therefore, using propagation of error^2^

${S\varphi}_{CT}= \left( \frac{{\partial\varphi}_{CT}}{\partial a_{R}} \right)^{2}S_{{\delta a}_{R}}+\left( \frac{{\partial\varphi}_{CT}}{\partial a_{I}} \right)^{2}S_{{\delta a}_{I}}$ (S22)

With ${S\varphi}_{CT}$ the PSD of the phase noise.

The phase response of the resonator given by equation (S17) can be differentiated with respect to the components of the amplitude noise, as

$\frac{{\partial\varphi}_{CT}}{\partial a_{R}}=-\frac{a_{I}}{a_{R}^{2}+a_{I}^{2}}=\frac{1-\left| h \right| \cos\left( \phi_{PD} \right)}{4\Omega_{D}^{2}+1-\left| h \right|^{2}}\frac{1}{\left| a \right|^{2}}$ (S23)

$\frac{{\partial\varphi}_{CT}}{\partial a_{I}}=\frac{-2\Omega_{D}-\left| h \right| \sin\left( \phi_{PD} \right)}{4\Omega_{D}^{2}+1-\left| h \right|^{2}}\frac{1}{\left| a \right|^{2}}$ (S24)

Equations (S18), (S23) and (S24) can now be inserted into equation (S22), to obtain

${S\varphi}_{CT}= S_{{\delta a}_{R}}\left( \frac{\left( 1-\left| h \right| \cos\left( \phi_{PD} \right) \right)\left( 4\Omega_{D}^{2}+1-\left| h \right|^{2} \right)}{4\Omega_{D}^{2}+4\Omega_{D}\left| h \right| \sin\left( \phi_{PD} \right)+1+\left| h \right|^{2}-2\left| h \right| \cos\left( \phi_{PD} \right)} \right)^{2}+S_{{\delta a}_{I}}\left( \frac{\left( -2\Omega_{D}-\left| h \right| \sin\left( \phi_{PD} \right) \right)\left( 4\Omega_{D}^{2}+1-\left| h \right|^{2} \right)}{4\Omega_{D}^{2}+4\Omega_{D}\left| h \right| \sin\left( \phi_{PD} \right)+1+\left| h \right|^{2}-2\left| h \right| \cos\left( \phi_{PD} \right)} \right)^{2}$ (S25)

where equations (S19) and (S20) still need to be used.

III.1.3. Frequency noise

Assuming the frequency noise is a function of the phase noise, one can use propagation of error to write

$S_{\Omega}=\left( \frac{\partial\Omega_{D}}{{\partial\varphi}_{CT}} \right)^{2}S_{\varphi_{CT}}$ (S26)

Therefore, the PSD of the frequency noise results from dividing the phase noise, given by equation (S25), by the slope of the frequency response of the parametrically pumped resonator, as^2^

$S_{\Omega}^{1/2}=\frac{S_{\varphi_{CT}}^{1/2}}{\left( \frac{{\partial\varphi}_{CT}}{\partial\Omega_{D}} \right)}$ (S27)

Where the slope of the frequency response is given by the derivative of equation (S17), as

$\frac{{\partial\varphi}_{CT}}{\partial\Omega_{D}}=\frac{\partial}{\partial\Omega_{D}}\mathrm{arctg}\left[ \frac{\left( 1-\left| h \right| \cos\left( \phi_{PD} \right) \right)/2}{\Omega_{D}+\left( \left| h \right| \sin\left( \phi_{PD} \right) \right)/2} \right]=-\frac{\left( 1-\left| h \right| \cos\left( \phi_{PD} \right) \right)/2}{\left( \Omega_{D}+\frac{\left| h \right| \sin\left( \phi_{PD} \right)}{2} \right)^{2}+\left( \frac{1-\left| h \right| \cos\left( \phi_{PD} \right)}{2} \right)^{2}}$ (S28)

Equations (S25) and (S28) are substituted into equation (S27) to determine the frequency noise.

III.1.4. Amplitude, Phase and Frequency noise, function of $\phi_{PD}$

Fig. S4 is used to illustrate the noise terms discussed in the previous sections, for the case of $\Omega_{D}=0$ (microcantilever driven at its natural frequency).

The contents of this figure were previously shown and discussed in the work of Mohammadi et al^2^, from where this analytical model is adopted. Nevertheless, they are re-plotted here with the correction for the mentioned shift of $\pi/2$ in $\phi_{PD}$, and to help supporting the conclusions of the present work.


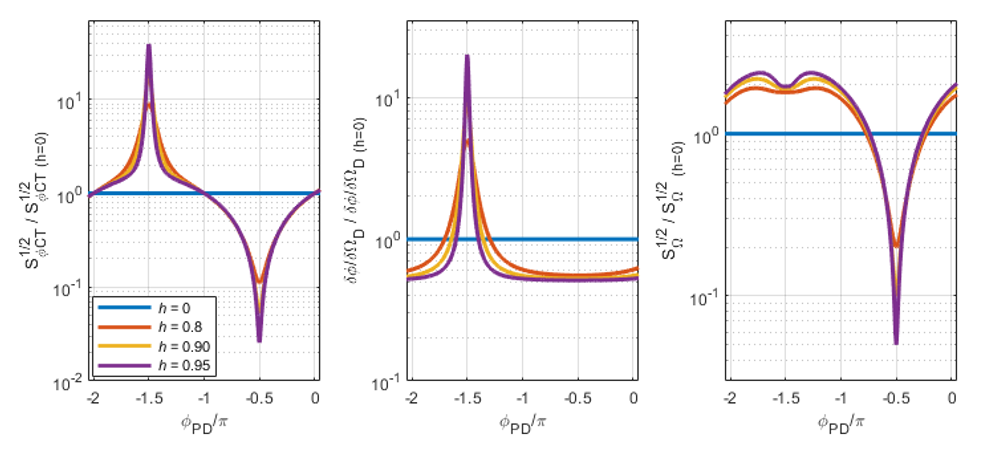


**Figure S4** PSD of the phase noise, slope of the resonator phase response and PSD of the frequency noise, as function of the parametric pumping phase ($\phi_{PD}$) and gain (*h*), for $\Omega_{D}=0$.

**III.2. Power Spectral Density of the in-phase component *X* in open-loop configuration**

Figs. S5 and S6 show histograms of the values of *X* measured in open-loop and closed-loop PLL configurations, respectively. Fig. S5 presents the results from the experiment described in Fig. S3 and in Fig. 4 of the main document, while Fig. S6 presents the results from the experiment described in Fig. S2 and in Fig. 3 of the main document. A fit of the normal distribution to the values of *X* is used to calculate the standard deviation of the distribution as function of the parametric gain and phase $\phi_{PD}$.


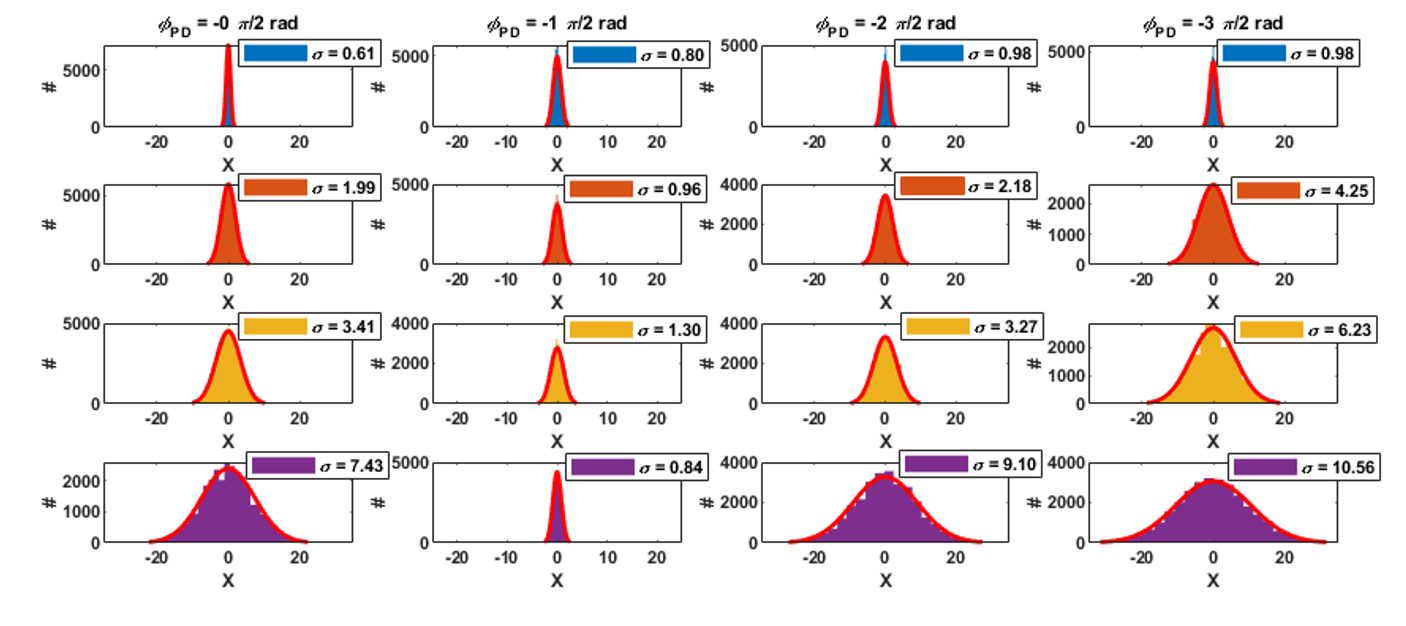


**Figure S5** Histogram of *X-*signal measured in open-loop as function of the parametric gain and phase. The parametric gain increase along the column, from zero (blue) to ~0.90 of the threshold value (purple). A fit of the normal distribution is shown in red, with the standard deviation of the distribution in the legend.


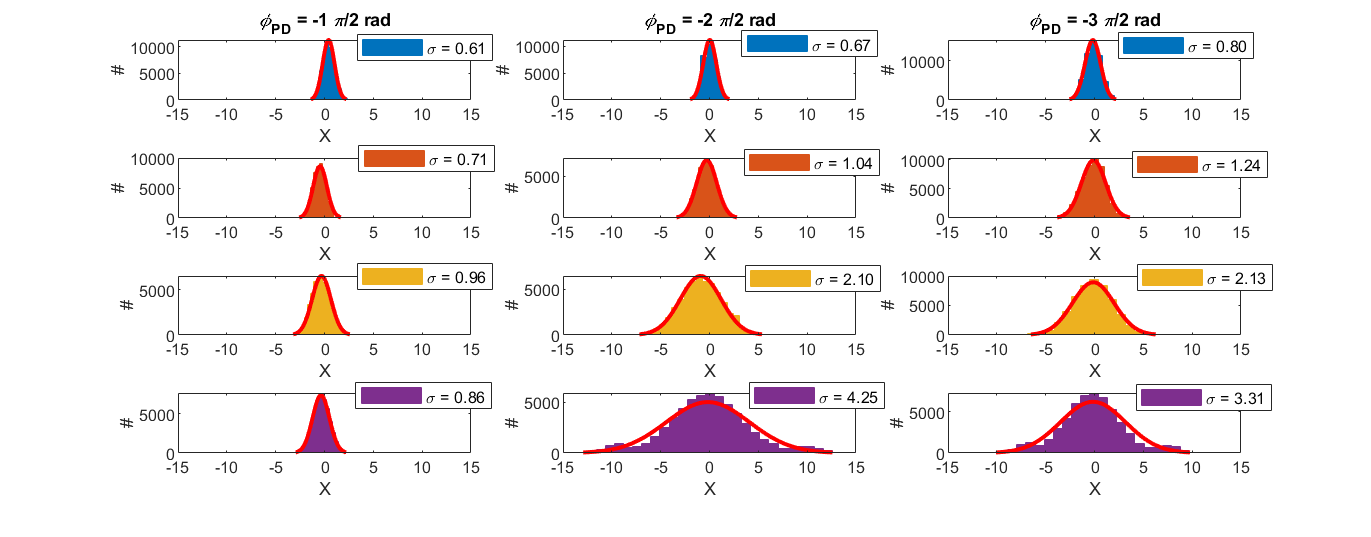


**Figure S6** Histogram of *X-*signal measured in closed-loop PLL (see Fig. S2) as function of the parametric gain and phase. The parametric gain increases along the column from 0 (zero) to ~0.80 of the threshold value (purple). A fit of the normal distribution is shown in red, with the standard deviation of the distribution in the legend.

In both figures it can be observed that the standard deviation of the distribution increases with the parametric pump gain (blue to purple) in all cases of $\phi_{PD}$, except for $\phi_{PD}=-1\pi/2 \mathrm{rad}$, as discussed and modelled in the right panel of Fig. 5 in the main document.

As indicated in Fig. S3, the noise-driven deflection of the cantilever can be divided into quadrature phases, as^6,7^

$A(t) \sin\left( \omega_{0}t+\varphi_{CT}(t) \right)=X_{1}\left( t \right)\cos\left( \omega_{0}t \right)+X_{2}\left( t \right)\sin\left( \omega_{0}t \right)$ (S29)

where $X_{1}\left( t \right)$ and $X_{2}\left( t \right)$ are random variables that vary slowly compared to $\omega_{0}$, caused by the noise in the amplitude and phase of the deflection. After demodulating the deflection of the cantilever by the reference signals, and considering $\phi=0$ for simplification, *X* is given by

${X(t)=X}_{1}\left( t \right)=A(t) \sin\left( \varphi_{CT}(t) \right)$ (S30)

Writing *X* as a function of the amplitude and the phase of the cantilever, such as $X(A,\varphi_{CT})$, propagation of error can be used, leading to

$S_{X}= \left( \frac{\partial X}{\partial A} \right)^{2}S_{{\delta a}_{R}}+\left( \frac{\partial X}{{\partial\varphi}_{CT}} \right)^{2}S_{\varphi_{CT}}$ (S31)

Where $S_{X}=\sigma_{X}^{2}$ is the variance of the *X* values. The PSD of the *X* signal is obtained by considering the PSD of the real part of the amplitude noise, given by equation (S15) and the PSD of the phase noise given by equation (S25). The PSD calculated with equation (S31) is plotted in Fig. 5 of the main document and compared with the standard deviation of the values of *X* measured experimentally and shown in Fig. S5.

**References**

1. Lifshitz, R. & Cross, M. C. Nonlinear Dynamics of Nanomechanical and Micromechanical Resonators. *Rev. Nonlinear Dyn. Complex.* 1–52 (2009) doi:10.1002/9783527626359.ch1.

2. Mohammadi, Z. *et al.* On the effect of linear feedback and parametric pumping on a resonator’s frequency stability. *New J. Phys.* **22**, (2020).

3. Karabalin, R. B., Masmanidis, S. C. & Roukes, M. L. Efficient parametric amplification in high and very high frequency piezoelectric nanoelectromechanical systems. *Appl. Phys. Lett.* **97**, 1–3 (2010).

4. Sader, J. E. Frequency response of cantilever beams immersed in viscous fluids with applications to the atomic force microscope. *J. Appl. Phys.* **84**, 64–76 (1998).

5. Maali, A. *et al.* Hydrodynamics of oscillating atomic force microscopy cantilevers in viscous fluids. *J. Appl. Phys.* **97**, (2005).

6. Miller, J. M. L. *et al.* Effective quality factor tuning mechanisms in micromechanical resonators. *Appl. Phys. Rev.* **5**, (2018).

7. Rugar, D. & Grütter, P. Mechanical parametric amplification and thermomechanical noise squeezing. *Phys. Rev. Lett.* **67**, 699–702 (1991).
